# Effectiveness of a nurse-led telephonic brief intervention to reduce risky alcohol use among women in primary care in Brazil: a randomized controlled trial

**DOI:** 10.1007/s00737-026-01772-5

**Published:** 2026-10-02

**Authors:** Ana Vitória Corrêa Lima, Jéssica Lima De Oliveira Pinheiro Gaia, Divane De Vargas

**Affiliations:** School of Nursing, Universidade de São Paulo, São Paulo, Brazil

**Keywords:** Brief intervention, Alcohol use, Women’s health, Randomized controlled trial, Primary health care, Telemedicine

## Abstract

**Background:**

Despite the increase in alcohol use among women worldwide, evidence on scalable, gender-responsive interventions remains limited, particularly in primary-care settings. This study evaluated the effectiveness of a nurse-delivered, telephonic brief intervention (BI) for reducing risky and harmful alcohol use among women.

**Methods:**

This randomized controlled trial included 151 women recruited from four primary-care facilities in São Paulo, Brazil, who scored 8–19 on the Alcohol Use Disorders Identification Test (AUDIT). Participants were randomly assigned to an intervention group (IG), which received a nurse-delivered telephone BI, or a control group (CG), which received feedback only. The primary outcome was the change in AUDIT scores assessed longitudinally from baseline through 24 months of follow-up.

**Results:**

In women receiving the telephonic BI, AUDIT scores significantly reduced during the first 6 months postintervention. Although this benefit was attenuated over longer-term follow-up, AUDIT scores in the control group followed an increasing trajectory over time. Race and household income were significantly associated with AUDIT scores; this suggests that socioeconomic factors may influence patterns of alcohol-related risk and intervention outcomes.

**Conclusions:**

A nurse-delivered telephonic BI produced meaningful short- to medium-term reductions in risky and harmful alcohol use among women in the primary-care setting. The attenuation of effects beyond 6 months suggests that a single brief intervention may be insufficient to sustain behavioral change over the long term and supports the evaluation of booster sessions or other low-intensity follow-up strategies. As a low-cost and scalable approach that can be integrated into routine primary care, telephonic BI may expand access to alcohol-related preventive care for women, particularly in resource-constrained settings.

**Clinical trial number:**

RBR-6msv2kv

## Introduction

Historically, men have tended to consume more alcohol than women. However, this trend has shifted over the years, with an increasing number of women consuming alcohol. According to the World Health Organization, the proportion of women who consume alcohol has increased worldwide, which has contributed to a narrowing of the gender gap, with projections indicating comparable consumption levels by 2030 (World Health Organization 2024). In the Americas, this trend is even more pronounced, with more than half (51.9%) of women reporting alcohol consumption (World Health Organization 2018; Costa et al. 2024).

Biologically, women are more vulnerable to the effects of alcohol than men, which makes them more prone to developing alcohol-use disorders at an earlier age. In addition to physiological factors, women frequently use alcohol as a coping mechanism for stress, trauma, emotional distress, anxiety, mood disorders, and to deal with the overload of work and responsibilities related to household or motherhood (Hughes et al. 2020; Hahm et al. 2023; Lunnay et al. 2023; Kersey and Lyons 2025). Furthermore, social factors influence alcohol consumption among women as does the change in their roles over the years. Women are increasingly present in the labor market and take on more responsibilities within their familial contexts and sometimes serve as the primary breadwinners. Even with these changes, there is a prevailing belief that women should not drink or have no need to drink, which may discourage them from seeking health services when experiencing alcohol-related issues (Finn et al. 2023).

Despite the magnitude of its impact on women’s lives, harmful alcohol use, defined as a use that impacts one’s physical, mental, or social health, is not reported by women frequently as a primary complaint in healthcare settings and is instead identified through indirect manifestations, such as anxiety, depression, insomnia, nonspecific physical symptoms, gastrointestinal and cardiovascular problems, and reproductive health issues. Psychosocial factors, including family conflict and violence, are associated with this pattern, often with alcohol use constituting a coping strategy. This contributes to under-reporting and hinders early identification of harmful alcohol use in healthcare services (Soares et al. 2020).

Because women who seek healthcare services frequently report diverse and nonspecific complaints and do not associate these symptoms with alcohol consumption, healthcare professionals must be trained to identify patterns of harmful alcohol use and implement interventions that are aimed at reducing consumption and related harms. In this context, primary health care (PHC), as the main entry point to the healthcare system, plays a key role in the early identification and management of alcohol-related problems among women (Gonzalez et al. 2020; Madhombiro et al. 2020; Kamal 2020; Lock and Kaner 2004; Gryczynski 2021; Gebara et al. 2013; Instituto Brasileiro de Geografia e Estatística 2021). Among the preventive strategies recommended for PHC, Screening and Brief Intervention (SBI) has been highlighted by the WHO as an effective approach for identifying and addressing risky alcohol use (Soares and Vargas 2020; Gonzalez et al. 2020; Madhombiro et al. 2020; Kamal 2020; Lock and Kaner 2004; Gryczynski 2021; Gebara et al. 2013; Instituto Brasileiro de Geografia e Estatística 2021; Platt et al. 2016; Babor and Higgins-Biddle 2001).

Developed by Sanchez-Craig and colleagues in the 1970s, the Brief Intervention (BI) aims to promote behavioral change to reduce alcohol consumption by incorporating principles of Motivational Interviewing (Harris et al. 2009). It is structured around six components that are summarized by the acronym F.R.A.M.E.S.: Feedback, Responsibility, Advice, Menu of Options, Empathy, and Self-efficacy, which remain active throughout the intervention (Babor and Higgins-Biddle 2001).

BI can be delivered either face-to-face or through information and communication technologies (ICTs), both showing effectiveness in reducing alcohol consumption, particularly among pregnant and postpartum women (Babor and Higgins-Biddle 2001; McKetta et al. 2023; Navarro et al. 2023; Alvanzo et al. 2011). In low- and middle-income countries such as Brazil, telephonic BI is a feasible and cost-effective strategy due to the widespread availability of mobile phones and internet services, with most households having access to them (Gebara et al. 2013; Instituto Brasileiro de Geografia e Estatística 2021). Furthermore, any health professional can delivery BI, especially nurses, who are on the front lines and are sometimes the professionals with the most contact with patients (Gonzalez et al. 2020). Nurses are in a strategic position to conduct brief interventions, and studies show that brief interventions delivered by nurses are effective and have positive impacts on the behavior changes related to reduction on alcohol consumption (Madhombiro et al. 2020, Kamal 2020; Lock and Kaner 2004; Gryczynski 2021). A systematic review showed that nurse-delivered BI was most effective in reducing alcohol consumption (Platt et al. 2016).

This study was conducted to evaluate the effectiveness of nurse-delivered, telephonic BI in reducing women’s harmful alcohol use. We hypothesized that, compared to feedback only, the intervention would lead to greater reductions in harmful drinking at 3, 6, and 24 months of follow-up.

## Methods

This was a single-blind, two-armed randomized controlled trial.

### Enrollment, screening, and randomization

#### Enrollment and eligibility

Participants were recruited through the appointment schedule of four PHC facilities in the central region of the city of São Paulo, Brazil. The study sites were strategically selected based on the high population density in the city’s central region, thereby enhancing the feasibility of participant recruitment.

### Data collection

From June 2021 to February 2022, monthly appointment schedules were provided by the participating PHC facilities. Participants were recruited via telephonic interviews within 3 days of their medical appointments at the PHC facilities and invited to participate in the study. Regardless of the type of consultation, clinical or otherwise, all participants were invited. The inclusion criteria were: age ≥ 18 years, having an appointment scheduled in a PHC facility, scoring between 8 and 19 in the Alcohol Use Disorders Identification Test in the interview (AUDIT), having a telephone line, and agreeing to participate in the study. Participants receiving any treatment for psychoactive substance use disorder at the time of data collection were excluded to avoid bias, as well as pregnant women, women with hearing impairments, those with difficulties with the national language (Brazilian Portuguese), and those who refused to receive the intervention.

The interviews were conducted by nine trained nurses. Before data collection, all nurses participated in a 20-hour structured training program delivered by the authors. The training consisted of weekly remote synchronous sessions, lasting approximately two hours each, conducted via the Google Meet platform. The training addressed the appropriate use of the online platform for data collection, procedures for accurately completing the study instruments, strategies for approaching participants by telephone, and standardized procedures for conducting the Brief Intervention and Feedback sessions only. The nurses were responsible for participant recruitment and intervention delivery. However, follow-up assessments were conducted by a different professional who was blinded to participants’ group allocation and had no access to the randomization information.

### Screening instruments

To characterize the sample, a questionnaire for sociodemographic and clinical characterization was developed by the authors and included questions on age, sexual orientation, race/ethnicity, marital status, religion, educational level, current occupation, housing situation, household income, and physical activities. Clinical variables that were assessed included hypertension, diabetes, mental disorders such as generalized anxiety disorder, depression, schizophrenia, bipolar disorder, eating disorders, personality disorders, mental health treatments, suicidal thoughts, psychiatric medications, behavioral variables, smoking, and illicit drug use, and questions regarding alcohol-consumption preferences, focusing on which type of alcoholic beverage was most frequently consumed by the participants.

The AUDIT was used to assess patterns of alcohol consumption. Standard drink sizes were clearly defined for participants when administering the quantity and frequency items, based on internationally recognized guidelines, to ensure consistent reporting. The minimum score was 0, and the maximum was 40, divided into four zones (I, II, III, and IV). Each zone represents a different consumption pattern: (a) Zone I: scores range from zero to seven, indicating low-risk use; (b) Zone II: scores from eight to 15, indicating a risky pattern, that does not yet cause harm; (c) Zone III: scores from 16 to 19, indicating harmful use, that already has impacts on one’s either physical or mental or social health; and (d) Zone IV: scores above 20, suggesting a probable dependence. According to the WHO, brief interventions should be conducted for those scoring in Zones II and III (Lima et al. 2024). The AUDIT has been validated in Brazilian Portuguese (Lima et al. 2005; Santos et al, 2012; Hopewell et al. 2025). Participant screening, randomization, and the interventions (BI or Feedback only) occurred on the same call.

### Randomization

Participants were randomly allocated to one of the two groups: the Intervention Group (IG) and the Control Group (CG). Randomization and allocation of participants were computer-generated, conducted using REDCap without researcher interference and before delivering the interventions. The research team first assessed participants for eligibility and obtained informed consent. Only after eligibility had been confirmed and consent obtained was the participant entered into the randomization procedure. Allocation was concealed until randomization was completed, as the researchers had no prior access to or knowledge of the allocation sequence and could not predict or influence the group to which a participant would be assigned. Participants were blinded to their group assignment. After randomization, researchers proceeded with the intervention according to the assigned groups. The randomization ratio of participants among the groups was 1:1.

### Intervention group

#### Brief Intervention

Those who were allocated in the intervention group (IG) received a single section of Brief Intervention based on the protocol developed by Lima et al. (Lima et al. 2024) and the following steps of this protocol:1) Feedback in which the participant reflects on alcohol use and its health impact; 2) Responsibility, whereby the participant assumes responsibility for change her alcohol consumption; 3) Advice, including guidance on risks, benefits of reducing, and harm reduction; 4) Menu of options, involving identification of risk and protective factors and behavior-change strategies that could be adapted according to the participant`s routine; 5) Empathy, characterized by a non-judgmental and non-confrontational approach; and 6) Self-efficacy, focused on reinforcing prior changes and the capacity for improvement. The intervention session had an average duration of 10 min.

### Control group

#### Feedback only

Participants in the CG received feedback, indicating that their alcohol consumption exceeded that recommended by the World Health Organization and it was advisable to reduce it. However, no detailed information on recommended doses, quantities, strategies or materials for reducing consumption was provided.

### Follow-ups interviews

Follow-up interview calls were conducted at prescribed intervals for both groups (IG and CG) after 3, 6, and 24 months. The calls were scheduled at the most convenient time for the participant to ensure privacy and to increase the retention rate. During the follow-up calls, only the AUDIT was administered.

### Primary outcome

Data were collected at baseline (T_0_), 3-months (T_1_), 6-months (T_2_) and 24-months (T_3_). The primary outcome was the relationship between AUDIT scores throughout the time measured during the three periods.

### Secondary outcomes

The secondary outcome was to evaluate the relationship between AUDIT scores and study variables, intention to become pregnant, sexual orientation, race, marital status, education, occupation, household income, welfare programs, hypertension, diabetes, cholesterol, gastritis/gastric problems, mental disorders, suicidal thoughts, smoking, and illicit drugs.

### Sample size

Power analysis indicated that to detect an effect size (f = 0.2948839) in the interaction between groups (control and experimental) and time interaction in a repeated measures design, revealed that a total sample of 24 participants would be sufficient to achieve 95% statistical power (Type II error: β = 0.05) while maintaining the significance level at 5% (Type I error: α = 0.05).

### Data analysis

Descriptive statistics were used to summarize sociodemographic, clinical, and behavioral characteristics, including means and standard deviations for continuous variables and absolute and relative frequencies for categorical variables. Longitudinal changes across the four assessment time points (T0, T1, T2, and T3) were analyzed using linear mixed-effects models (LMMs). Participants with incomplete follow-up were not excluded from the longitudinal analyses, as the LMM incorporated available repeated measurements across assessment time points. The analyses therefore included available outcome data from participants with incomplete follow-up rather than being restricted to complete cases. A first-order autoregressive covariance structure [AR(1)] was specified to account for the within-participant correlation among repeated measurements over time. Statistical significance was set at *p* ≤ 0.05. For the secondary outcome, the Fisher’s exact or Pearson’s chi-square test was applied, as appropriate according to the characteristics and distribution of the variables.

## Results

**Fig. 1:**
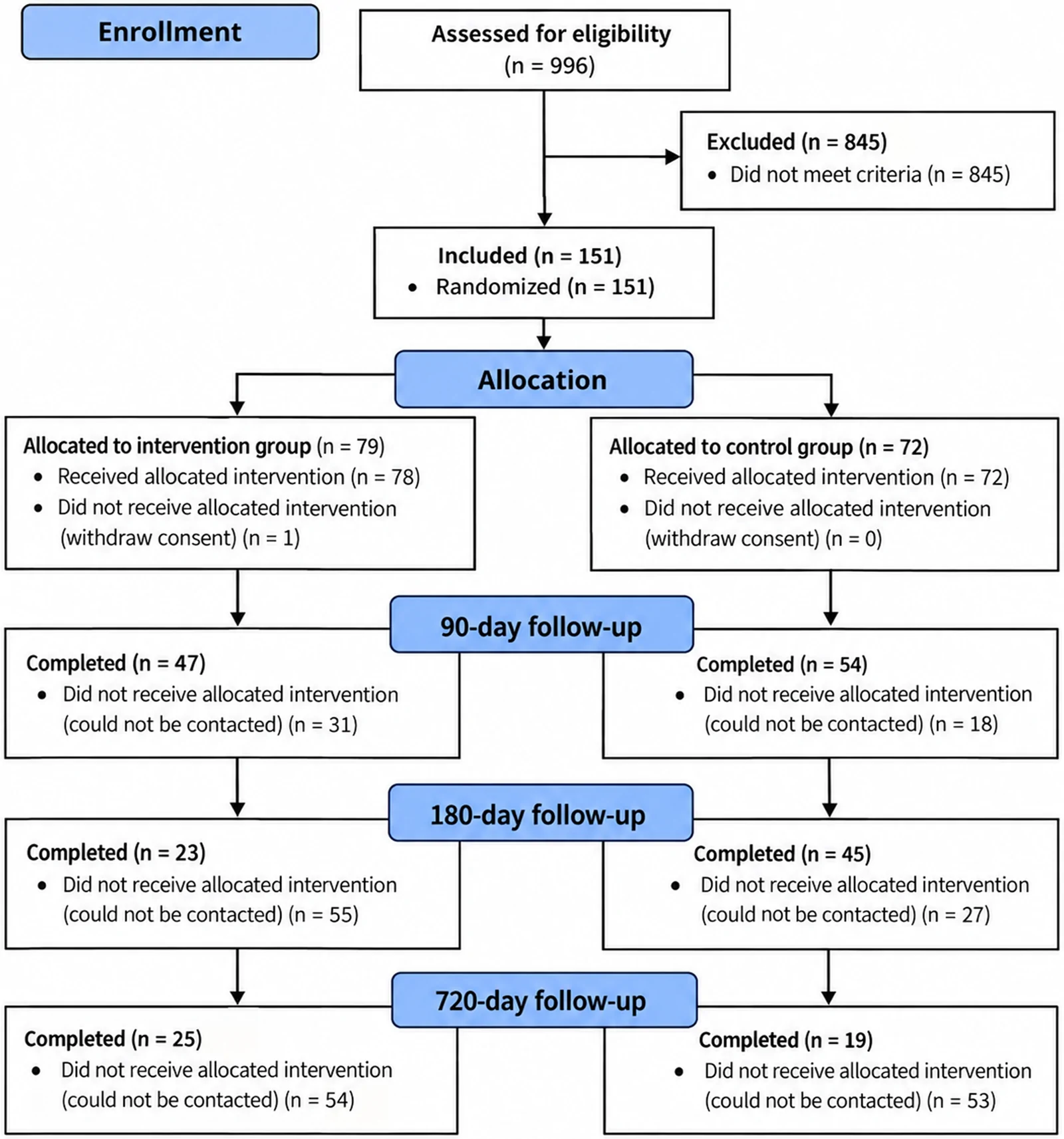
CONSORT flowchart, 2026

Across the four participating facilities, 1.132 women were assessed for eligibility, the mean of participants recruited per primary health care center was 283 women. Of those, 996 (88.3%) agreed to participate in the study, of whom 151 were eligible, randomized, and allocated to the intervention (*n* = 79) and control (*n* = 72) groups. The mean age of the included women (*n* = 151) was 32.8 years, white (53.6%), heterosexual (82.7%), single (60,9%), and employed (64.2%). Depression (24.5%) and anxiety (22.5%) were the most common self-reported mental health conditions. Baseline sociodemographic and clinical characteristics were balanced between the intervention and control groups participants, with no significant differences observed between both groups. This comparability was maintained throughout the follow-up assessments, with no significant changes in participant characteristics over time. Table 1 shows the baseline social and clinical characteristics of the participants (Fig. 1).

**Table 1:** Socio-clinical characteristics of participants, 2026

Variable	Level	Groups			
		Control		Intervention	
		N	%	N	%
Desire to Conceive	I am fine that way	11	15.2	13	16.4
	I am not sure about it	5	6.9	6	7.6
	Not now, but at some point, in the future	23	32	29	36.7
	No, I never intend to conceive	32	44.4	31	39.2
	Yes, I do	1	1.3	0	0
Sexual Orientation	Heterosexual	62	86.2	63	79.9
	Non-heterosexual	10	13.8	16	20.1
Race	White	32	44.4	49	62
	Brown	23	31.9	23	29.1
	Black	15	20.8	7	8.8
	Indigenous	2	2.7	0	0
Marital status	Single	44	61.3	48	61
	Married	24	33.2	20	25.2
	No longer in a relationship	4	5.5	11	13.8
	Elementary School	4	5.5	6	7.6
	High School	33	45.7	34	43.5
	Undergraduate	31	43	33	42.3
	Postgraduate	4	5.8	5	6.6
Occupation	Employed	41	56	56	70.8
	Unemployed	18	25	14	17.7
	Housewife	2	2.7	2	2.5
	Other	5	6.9	4	5
	Student	6	8.3	3	3.8
Household Income (Brazilian minimum wage: R$ 1,412 OR U$280)	1	17	26.1	9	12.1
	1–2	17	26.1	36	48.6
	2–5	26	40	23	31
	5–10	4	6.1	6	8.11
	10–20	1	1.5	0	0
Welfare Program	No	54	75	59	74.6
	Yes	18	25	20	25.3
Hypertension	No	65	90.2	68	86
	Yes	7	9.7	11	13.9
Diabetes	No	70	97.2	77	97.4
	Yes	2	2.7	2	2.5
Increased Cholesterol	No	65	90.2	71	89.8
	Yes	7	9.7	8	10.1
Gastritis/gastric Problems	No	60	83.3	59	74.6
	Yes	12	16.6	20	25.3
Generalized Anxiety Disorder	No	59	81.9	58	73.4
	Yes	13	18.0	21	26.5
Depression	No	54	75	60	75.9
	Yes	18	25	19	24
Schizophrenia	No	72	100	79	100
Bipolar Disorder	No	70	97.2	76	96.2
	Yes	2	2.7	3	3.8
Eating Disorders	No	71	98.6	73	92.4
	Yes	1	1.3	6	7.6
Personality Disorders	No	71	98.6	79	100
	Yes	1	1.3	0	0
Mental Health Treatment	No	47	65.2	56	70.8
	In the past	5	6.9	3	3.8
	Yes	20	27.7	20	25.3
History of Suicidal Behavior	No	51	70.8	61	77.2
	Yes	17	23.6	15	18.9
	I have attempted	4	5.5	3	3.8
Psychotropic Drug Use	No	55	76.3	60	75.9
	In the past	4	5.5	4	5
	Yes	13	18	15	18.9
Smoking	No	62	86.1	66	83.5
	Yes	10	13.8	13	16.4
Illicit Drug Use	No	65	90.2	69	88.4
	Yes	7	9.7	9	11.5

Table 2 presents the AUDIT scores for both groups across the four measurement periods. In the intervention group, the lowest AUDIT score was observed at T2, with a nearly 2-point difference compared to the other periods. In the control group, the lowest score was observed at T_0_. The intervention group showed a significant decrease, whereas the control group showed an increase, in AUDIT scores through the 180-day follow-up.

**Table 2:** Mean AUDIT scores for the control and intervention groups across the four assessment periods, 2026

Group	Time	*N*	Missing	Mean	SD	Min	Median	Max	Lower CI	Upper CI
Control	Screening	72	0	6.1	3.75	3	5	27	5.41	7.22
	Follow up 90	54	18	7.41	4.15	3	7	21	6.47	8.71
	Follow up 180	45	27	6.8	3.56	3	7	20	5.94	8.05
	Follow up 720	19	53	7.9	4.94	3	8	24	6.26	10.92
Intervention	Screening	78	1	7.83	4.74	3	7	28	6.93	9.05
	Follow up 90	47	31	5.75	2.89	3	5	17	5.05	6.72
	Follow up 180	23	55	5.39	2.62	3	4	13	4.54	6.73
	Follow up 720	25	53	9.16	6.1	3	7	26	7.24	12.09

Analysis of deviance revealed significant temporal dynamics and interaction effects (Table 3). Although the group variable alone showed no significant effect (*p* = 0.666), both time (*p* < 0.001) and the interaction between group and time (*p* < 0.001) were highly significant. This interaction (Group and Time) suggests that temporal patterns varied across the groups, implying that the intervention effect changed over time. These findings underscore the importance of considering temporal and group differences in future analyses.

**Table 3:** Analysis of deviance table (Type III Wald chi-square test)

	Chisq	Df	Pr(> Chisq)
(Intercept)	569.1	1	< 0.001***
Group	0.07	1	0.790
Time	16.4	3	< 0.001***
Group: Time	22.9	3	< 0.001***

The results demonstrated that, among all variables explored, only race (*p* = 0.035) and household income (*p* = 0.024) were significantly associated with variations in AUDIT scores, which suggests the influence of socioeconomic factors on alcohol-consumption patterns among women.

## Discussion

This study investigated the effectiveness of a nurse-led telephonic Brief Intervention (BI) for reducing risky and harmful alcohol use. The results indicated that the participants were predominantly young women (mean age 32.8 years), white, heterosexual, single, and economically active. This profile is consistent with recent findings, in the international literature, of an increase in alcohol consumption among women that is associated with the social, cultural, and gender transformations in contemporary society. Alcohol consumption among women has been linked to greater female autonomy, the occupation of new spaces, greater social pressure, and the pursuit of freedom and independence (Hahm et al. 2023; Lunnay et al. 2023; Kersey and Lyons 2025; McKetta et al. 2023; Navarro et al. 2023; Alvanzo et al. 2011; Mulia and Bensley 2020).

This cohort comprised a high proportion of women with reproductive potential, and approximately 40% of the women in the intervention group reported future reproductive plans to conceive. This finding deserves attention because alcohol consumption during pregnancy is associated with significant maternofetal risks, such as premature birth and fetal malformations, and maternal abstinence is recommended during pregnancy. Furthermore, even before conception, alcohol consumption can vary according to fertility (Heertum and Rossi 2017; Fan et al. 2017). These findings highlight the need for more research on alcohol consumption among women who plan to conceive because early interventions can help improve fertility, reduce the risk of pregnancy-related complications or fertility problems, and serve as a motivational factor for reconsidering alcohol consumption.

Beyond reproductive aspects and individual behavioral factors, the findings highlighted the influence of social determinants on alcohol consumption patterns among women. Race and household income were associated with AUDIT scores, which reinforced the role of socioeconomic and racial inequalities in shaping alcohol-use behaviors. The majority of participants were white women; previous studies have reported similar results, with white women having a higher prevalence of alcohol use-related problems than women of other races (Black, Hispanic, and Asian) (Navarro et al. 2023, Alvanzo et al. 2011, Mulia and Bensley 2020). This might be explained by the inequalities and difficulties in access to health services for women of color and the bias of healthcare providers in assessments of alcohol use among those women. In the Brazilian context, inequalities are associated with greater exposure to stressors, violence, geography, emotional burden, limited access to healthcare services, and psychological distress, all of which may contribute to harmful alcohol use. Sociodemographic characteristics, including income, race, social vulnerability, and psychosocial conditions, significantly influenced alcohol-consumption patterns and alcohol-related harms among individuals (Alvanzo et al. 2011; Mulia and Bensley 2020; Chinneikim et al. 2024; Heertum and Rossi 2017; Fan et al. 2017; Kilian et al. 2023; Cardoso et al. 2025).

Our findings indicate that the trained nurse-delivered BI effectively reduced women’s risky and harmful alcohol use. A nurse-delivered BI enhanced participants’ motivation, which may have influenced their alcohol consumption (Chinneikim et al. 2024). This might be related to the fact that nurses are frontline healthcare professionals and tend to develop closer relationships with patients than other professionals. These relationships may positively influence patients’ perceptions and help them engage more actively in behavioral change. With appropriate support from healthcare managers and service leaders, nurses and others health professionals can play an important role in the continuous delivery of BIs. Therefore, managers should invest in strengthening their implementation and develop strategies to integrate BIs into routine care and nurses’ and others professional’s daily activities.

The intervention effects were sustained for up to 180 days, a period during which participants presented the lowest AUDIT scores. However, after this point, AUDIT scores increased, which suggests a gradual reduction in the maintenance of the intervention effects over time in a pattern similar to that observed in prior investigations (Woliansky et al. 2024; Yonkers et al. 2020, Giménez-Costa et al. 2025; Kuntsche et al. 2017). An extra session at 180-days could increase the effect of the intervention for longer periods and increase the rotation rate of the participants (Cardoso et al. 2025; Plens et al. 2022; Woliansky et al. 2024). Further studies are needed to assess whether delivering either a booster intervention after 180 days or one or more sessions before 90 and 180 days maintains the observed benefits over a longer period.

No withdrawals were documented as being directly related to the intervention; losses to follow-up occurred primarily because participants could not be reached for the scheduled assessments. Despite repeated contact attempts, telephonic follow-up was unsuccessful in several cases owing to unanswered calls, changes in participants’ telephone numbers, or disconnected or inactive telephone lines. This challenge should be considered within the Brazilian context, where the use of prepaid mobile phone services is widespread and users may frequently change telephone numbers or SIM cards, including to take advantage of promotional benefits that are offered with new prepaid lines. Consequently, maintaining stable telephonic contact over an extended follow-up period can be particularly challenging in this population. Thus, most losses appeared to reflect difficulties in maintaining contact with participants over time rather than active withdrawal from the study. Importantly, no participant withdrawal was documented as being directly attributable to the intervention or to intervention-related adverse experiences.

The components of the intervention group were based on the FRAMES model, which is grounded in Motivational Interviewing and the Transtheoretical Model. Previous studies suggested that additional BI booster sessions may influence motivation, self-efficacy, coping strategies, and relapse-prevention skills, and thus potentially support the maintenance of alcohol-consumption reduction among women over time (Kilian et al. 2023, Cardoso et al. 2025, Plens et al. 2022, Woliansky et al. 2024, Yonkers et al. 2020, Giménez-Costa et al. 2025, Kuntsche et al. 2017; McGettrick 2024; Carey et al. 2024). Research is needed to ascertain whether the length of the booster intervention should be the same, shorter than, or longer than the previous BI.

Although the mean AUDIT scores of the control group was lower at baseline, throughout the study period, their alcohol consumption, especially at 90 and 720 days, increased; however, the control group showed a decrease in consumption at 180 days. Recent intervention studies have shown that providing individualized feedback to women can significantly influence their subsequent behaviors and self-reported responses. Feedback tailored to women-specific health risks frequently leads to modifications in both reported and actual alcohol consumption (Kuntsche et al. 2017; McGettrick 2024; Carey et al. 2024; Tan et al. 2023; Smith et al. 2023). Providing standardized brief feedback to both groups while reserving the active components of the BI for the intervention group enables a clearer assessment of the intervention effect. Nonetheless, it is unclear why the decrease occurred at 180 days and not as early as 90 days.

### Strengths and limitations

This study has notable strengths, including its unique contribution as the only published randomized controlled trial in Latin America that focuses on women and its systematic 2-year follow-up, which together enhance the reliability of its findings. However, some limitations must be acknowledged. The data collection occurred during the public health emergency of COVID-19, which may have influenced participant recruitment, data-collection procedures, and participants’ behaviors and responses, and thereby potentially impacted the study findings and their interpretation. The scarcity of evidence on how individuals perceive their own drinking patterns, particularly in Brazil, highlights an important area for future research on alcohol-related interventions. Although statistically significant reductions in AUDIT scores were observed, a threshold for clinically meaningful change was not predefined, which limits conclusions regarding the clinical significance of these changes. Clinical conditions were assessed only at baseline using dichotomous (yes/no) questions, which further limited our ability to evaluate whether reductions in alcohol consumption were accompanied by perceived or measurable improvements in participants’ health. Future studies should incorporate longitudinal assessments of women’s perceived health and validated measures of health-related quality of life, to better determine the clinical relevance of changes in alcohol consumption. Furthermore, the analytical approach may have limited a more detailed exploration of temporal and between-group differences, which should be further investigated. A key limitation was the loss to follow-up owing to difficulties in maintaining telephonic contact, particularly in a context where prepaid mobile users may frequently change phone numbers. This may have affected participant retention and introduced attrition bias. Future studies could mitigate this limitation by collecting multiple contact methods, including alternative phone numbers, email addresses, WhatsApp^®^, and contact information from a trusted person, and periodically updating participants’ contact details. Future research should aim to improve participant retention, integrate objective alcohol-use assessments, and test interventions in diverse settings to overcome these limitations.

## Conclusions

This study demonstrated the potential of a nurse-delivered telephonic BI to reduce risky alcohol use among women in the primary care setting. The intervention’s effects lasted up to 6 months, which underscores the need for ongoing support and potential booster sessions to sustain its benefits. The significant role of socioeconomic factors in alcohol-use patterns underscores the importance of tailoring interventions to address the unique needs and barriers faced by women from diverse backgrounds. Integrating this cost-effective, electronically delivered BI into public health policies could help address the growing problem of alcohol misuse among women and provide an effective strategy for targeted interventions.

## Data Availability

No datasets were generated or analysed during the current study.

## Abbreviations

AUDIT: Alcohol Use Disorders Identification Test
BI: Brief Intervention
CONSORT: Consolidated Standards of Reporting Trials
CG: Control group
ICTs: Information and communication technologies
IG: Intervention group
PHC: Primary Health Care
RCT: Randomized clinical trial
SBI: Screening and Brief Intervention
WHO: World Health Organization
